# Patient satisfaction and postoperative pain management in ambulatory surgery: a prospective questionnaire-based observational cohort study at a Tertiary University Hospital

**DOI:** 10.3389/fmed.2026.1862773

**Published:** 2026-07-02

**Authors:** Omar A. Ababneh, Ahmad I. El-Share’, Isam Bsisu, Abdulrahman Abu-Hamdan, Asma Zaid Alkilani, Yara Baher, Lara A. Alsaeedy, Reem M. Alhyari, Abdallah M. Elqunj, Ali Yaghi, Ahmad Shahin, Walid Samarah, Lubna A. Khreesha, Adel F. Alrabadi, Subhi Alghanem

**Affiliations:** 1 Department of Anesthesia and Intensive Care, School of Medicine, The University of Jordan, Amman, Jordan; 2 Department of Anesthesia and Pain Management, King Hussein Cancer Center, Amman, Jordan; 3 Department of Anesthesia and Perioperative Medicine, Schulich School of Medicine and Dentistry, Western University, London, ON, Canada; 4 Department of Special Surgeries, School of Medicine, The University of Jordan, Amman, Jordan

**Keywords:** ambulatory surgery, post op complication, post-anesthesia care unit, post-operative pain, satisfaction

## Abstract

**Introduction:**

Ambulatory surgery is expanding globally due to its clinical, social, and economic benefits, yet postoperative pain remains a major determinant of patient satisfaction and is often inadequately managed.

**Methods:**

This prospective questionnaire based observational cohort study at Jordan University Hospital (January–September 2024) evaluated patient satisfaction with ambulatory surgery and its association with postoperative pain control. Adult patients undergoing elective ambulatory procedures with preoperative anesthesia clinic attendance were included (*N* = 1012). Satisfaction was assessed using predefined 10-point Likert scales across perioperative domains, with pain intensity measured by the Numeric Rating Scale (NRS-11).

**Results:**

Overall satisfaction with anesthesia care was reported by 97.6% of the patients. However, dissatisfied patients reported significantly lower overall hospital satisfaction [median 7 (IQR 6–9) vs. 9 (IQR 8–9); *p* < 0.001] and reduced willingness to recommend the hospital (87.5% vs. 98.8%; p = 0.002). Overall satisfaction positively correlated with staff approach in the pre-anesthesia clinic, holding area, and post-anesthesia care unit, and with pain management (*p* < 0.001), while negatively correlating with postoperative pain scores (*p* < 0.001). Multivariable analysis identified better post-anesthesia care unit approach (OR 1.70, 95% CI 1.31–2.22) and regular pain assessment (OR 3.77, 95% CI 1.17–12.21) as independent predictors of satisfaction, whereas perceived under-treatment predicted dissatisfaction (OR 0.31, 95% CI 0.10–0.92).

**Conclusion:**

These findings underscore the critical role of proactive pain management and high-quality perioperative communication in improving patient satisfaction after ambulatory surgery.

## Introduction

Ambulatory surgery has become the dominant model for elective procedures worldwide, driven by advances in surgical techniques, anesthesia, and perioperative care that enable safe same-day discharge while reducing healthcare costs and improving patient experience (1, 2). In this evolving landscape, Enhanced Recovery After Surgery (ERAS) principles—originally developed for major inpatient procedures—have been successfully adapted to ambulatory settings, emphasizing multimodal analgesia, opioid-sparing techniques, early mobilization, and patient-centered care to optimize recovery and satisfaction (3, 4).

Modern ambulatory anesthesia now prioritizes proactive pain management through multimodal, opioid-minimizing strategies, including regional anesthesia, non-opioid analgesics (acetaminophen, NSAIDs, gabapentinoids), and adjuncts such as dexmedetomidine or lidocaine infusions. These approaches aim to reduce postoperative pain, opioid-related side effects, and delayed discharge while enhancing patient-reported outcome measures (PROMs) (5, 6).

Despite these advances, postoperative pain remains a major barrier to optimal recovery and a key determinant of patient satisfaction in day-case surgery (7–10). Patient satisfaction, increasingly recognized as a critical PROM, is influenced not only by pain control but also by perioperative communication, staff empathy, and alignment between patient expectations and delivered care. However, data from low- and middle-income settings, including the Jordanian healthcare system, remain limited, particularly regarding the integration of contemporary ERAS-aligned practices in ambulatory anesthesia.

This study aimed to evaluate patient satisfaction with ambulatory surgery at Jordan University Hospital and its association with postoperative pain management practices within the context of evolving perioperative standards.

### Study objectives

The study aimed to quantifying patient-centered outcomes in ambulatory anesthesia.

A- To evaluate the level of patient satisfaction with the ambulatory (day-case) surgical process at Jordan University Hospital (JUH).
B- To determine the correlation between patient satisfaction and the effectiveness/adequacy of postoperative pain control.

## Materials and methods

### Study design

This questionnaire-based observational cohort study was conducted at Jordan University Hospital (JUH); a tertiary referral and teaching institution in Amman-Jordan. We adhered to STROBE (11) reporting guidelines in the editing of this article. Data were collected over a 9-month period from January to September, 2024.

### Inclusion criteria

Patients were eligible for inclusion if they met the following criteria:

* Age more than or equals 18 years.
* Underwent an elective ambulatory (day-case) procedure, defined as a procedure with no planned overnight hospital stay.
* Attended a preoperative assessment at the pre-anesthesia clinic.
* Patients who received general anesthesia (including total intravenous anesthesia (TIVA) or volatile-based maintenance), neuraxial anesthesia (intra-thecal or epidural blocks), and loco-regional/peripheral nerve blocks with or without sedation.

### Exclusion criteria

Patients with any of the following were excluded from the sample:

* Aging less than 18 years.
* Declined to provide informed consent.
* Patients previously diagnosed with a cognitive impairment or a psychiatric disorder.
* Emergency and urgent procedures.
* Non-surgical interventions (e.g., endoscopies) and redo surgeries.

### Informed consent

All potential participants received a detailed written and verbal explanation of the study objectives and their right to withdraw at any time without any consequences. All participants provided an informed written consent prior to surgery. Clear contact details of the principal investigator were provided to the participants for any questions or concerns arising before or after enrollment.

### The questionnaire

The questionnaire consisted of two main sections and was administered by a trained investigator in Arabic.

*Section one*: Demographic, pre-operative and perioperative data.

This section collected socio-demographic characteristics and perioperative (anesthetic and surgical) details. It also included two satisfaction item rated on a ten point Likert scale (1 = extremely dissatisfied, 10 = extremely satisfied):

satisfaction with the approach and communication in the preoperative anesthesia clinic visit. and satisfaction with the approach at the surgical holding area.

Satisfaction with anesthesia care and other perioperative domains was assessed using 10-point Likert scales (1 = extremely dissatisfied, 10 = extremely satisfied). For the primary analysis, patients were dichotomized as “satisfied” (score ≥ 6) or “dissatisfied” (score < 6). This cutoff was chosen because it represents a clear threshold separating neutral-to-mildly positive responses from clearly positive ones, a convention frequently used in patient satisfaction research. This approach has been employed in multiple perioperative and anesthesia satisfaction studies (7, 12–14).

*Sections two*: Postoperative experience and pain management.

This section addressed postoperative adverse events and pain-related outcomes:

- Occurrence and timing of pain assessment in the post-anesthesia care unit’s (PACU), identity of the assessor and method of assessment.
- Patients’ perception whether analgesia was administered as prescribed.
- Patients’ feeling of being undertreated.
- Patient’s being treated as malingering or drug-seeking when requesting analgesia.

Pain intensity was recorded using the 11-point Numeric Rating Scale (NRS-11); (0 = no pain at all, 10 = the worst imaginable pain) at two time points: upon discharge from the PACU, and 6 h postoperatively.

Patients were discharged from PACU according to standard Aldrete score ≥ 9. (13) The 6-h postoperative pain assessment was performed exactly 6 h after PACU discharge while patients were on the surgical ward.

Patients’ satisfaction was recorded using the same 10-point Likert scale for the following items: satisfaction with pain management, satisfaction with anesthetic management, the overall satisfaction with hospital care, and whether they are going to recommend the hospital for others in the future. A score ≥ 6 is defined as satisfied.

### Validity, reliability, and sample size calculation

The questionnaire underwent content validation by seven consultant anesthesiologists, they agreed on item relevance, coherence, clarity and sufficiency. Adequacy was recorded quantitatively as the proportion of experts who agreed that the items were relevant, coherent, clear, and sufficient. Then, the research team modified the questionnaire based on their feedback.

Furthermore, we performed a pilot study, collecting data from 35 patients to assess for internal consistency. Internal consistency reliability was assessed for the satisfaction scale composed of perioperative satisfaction items (pre-anesthesia clinic, holding area, PACU approach, pain management satisfaction, and overall anesthesia satisfaction). Cronbach’s alpha in the pilot study was 0.72, indicating acceptable internal consistency. Comprehensive psychometric validation (e.g., factor analysis) was beyond scope.

The satisfaction items demonstrated coherent inter-item associations and behaved consistently with theoretical expectations, supporting construct validity.

Sample size was calculated assuming a satisfaction rate of 56.5% based on a previous investigation in our region (15), we calculated the representable sample size at 0.05 type I error rate and a 0.05 margin of error (16), which yielded a minimum sample size of 378 patients to achieve the desired level of precision.

### Data collection

The questionnaire was conducted via semi-structured interviews by second year anesthesia residents who were trained specifically for this study to conduct the survey under supervision from an anesthesia consultant. Plus, they were not involved in the patients’ direct clinical care, and conducted interviews in a standardized manner. The interview was conducted in Arabic language, which is the native language for both respondents and interviewees. Interviews were performed 6 h after the transfer to the floor from the PACU, prior to their discharge from the hospital.

Data validation was done using data triangulation method, where patient self-reporting, via the aforementioned questionnaire, was followed by reviewing hospital records to cross-check and to verify that the collected data was accurate, reducing the likelihood of biases associated with relying on a single data source.

Patients’ medical reports were reviewed for intraoperative critical incidents, patient-reported postoperative complaints and adverse events, documented pain scores, and analgesic drugs administration. Extracted data was checked against patients’ self-reported complaints in the questionnaire, and any events were in the medical record not mentioned by the patient were added to the dataset reducing the recall bias by patients forgetting or inaccurately recalling these events.

Pain scores were recorded primarily in the medical records in PACU and 6-h post-op. Patient reported scores were compared with scores documented in the medical record. Cases with a discrepancy greater than ++2 points on the NRS-11 were excluded from the final analysis to reduce reporting bias. We reduced the measurement bias, and confirmation bias by ensuring that all patients were asked the same questions at the same time and in the same way to evaluate their pain score. To reduce measurement and recall bias, cases with a discrepancy of more than ± 2 points between self-reported and documented pain scores were excluded from the primary analysis. However, a sensitivity analysis including all cases was conducted to evaluate the impact of this exclusion criterion on the primary findings.

### Study sample

Out of 1,250 patients screened, 238 were excluded for the following reasons: emergency/urgent procedures (*n* = 7), refusal of consent (*n* = 32), cognitive impairment (*n* = 3), non-surgical procedures (*n* = 126), and redo surgeries (*n* = 70). A total of 1,012 patients were included in the final analysis.

### Ethical statement and approval

The study was approved by Institutional Review Board (IRB) committee of Jordan University Hospital (approval number: 10/2023/7921) on 28 March 2023. A written informed consent was obtained at the beginning of the interview after explaining the primary aims of the study and the privacy of obtained information. We did not collect any identifying personal information, and collected data were blinded. The collected data was used solely for statistical analysis. No deviation from standard of care, and declining participants were guaranteed to face no consequences.

### Statistical analysis

Statistical analysis was performed using IBM SPSS Statistics version 25.0 (IBM Corp., Armonk, NY, United States). Descriptive statistics were used to summarize the data. Continuous variables are presented as median [interquartile range (IQR)], while categorical variables are expressed as frequency (percentage).

Between-group comparisons of continuous variables (e.g., age, pain scores, and satisfaction) were conducted using the Mann–Whitney U test. Categorical variables were compared using the chi-squared test or Fisher’s exact test, as appropriate. Given the near-continuous distribution and prior use of 10-point Likert scales in satisfaction research, Pearson’s correlation was considered appropriate and Pearson’s coefficients (r) were calculated to assess the strength and direction of linear relationships. Multivariable binary logistic regression analysis was employed to identify factors independently associated with dissatisfaction with anesthesia care. Results are reported as odds ratios (OR) with corresponding 95% confidence intervals (95% CI). A two-sided *p* < 0.05 was considered statistically significant for all tests.

The primary outcome was satisfaction with anesthesia care, dichotomized as satisfied (Likert score ≥ 6) or dissatisfied ( < 6).

## Results

**FIGURE 1:**
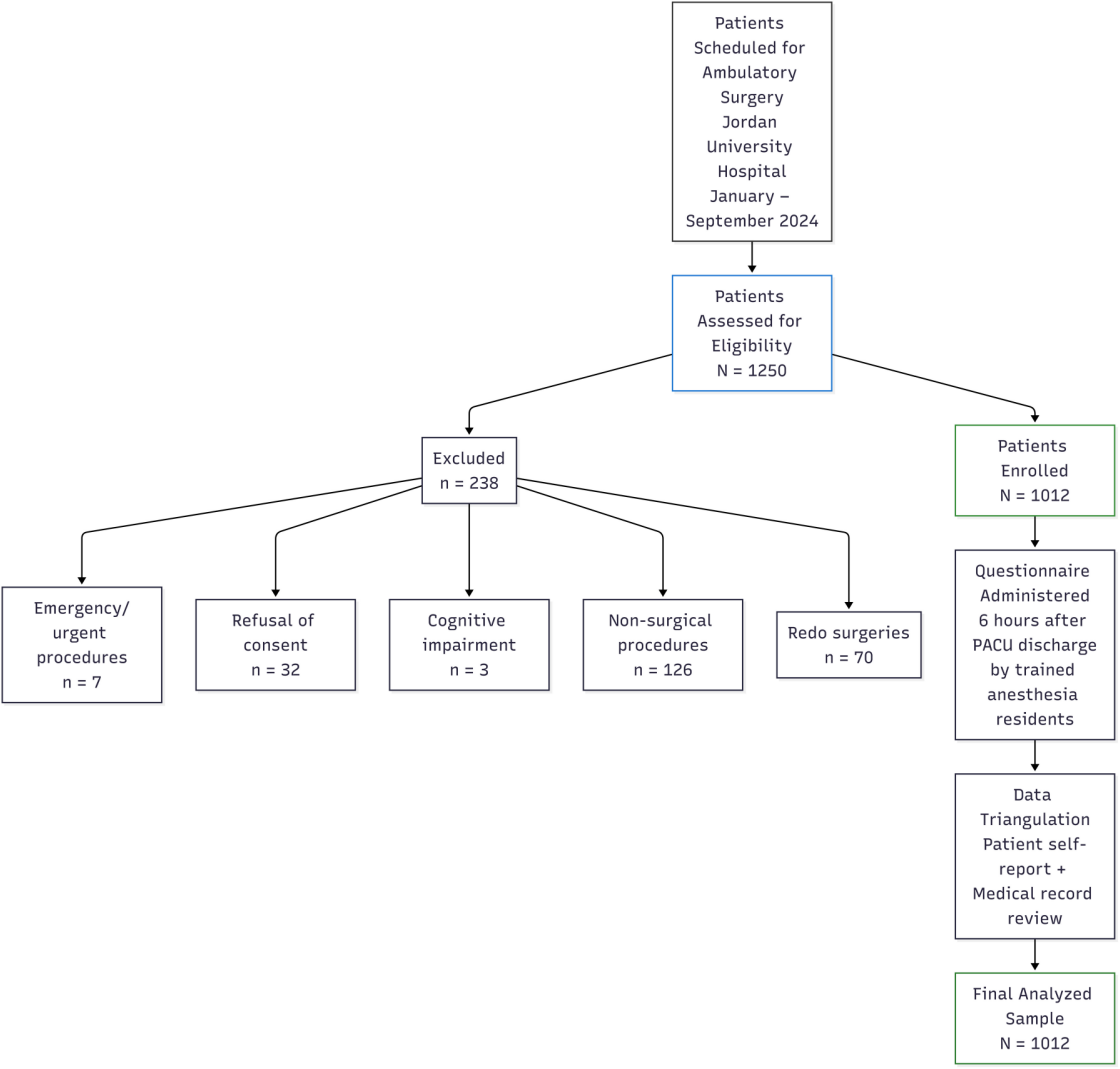
STROBE flow diagram of participant recruitment and selection in the study.

Overall, 1,012 patients were included in this study, with a median age of 45(31.3–60) years, of which 508 (50.2%) were males and 504 (49.8%) were females. The overall satisfaction with anesthesia care was 97.6%, since 988 patients were satisfied with anesthesia care. The predominant ASA Classification score was II, with 568 (56.1%) participants being ASA II patients, while 324 (32.0%) were ASA I patients. The most commonly used modality of anesthesia was general anesthesia, which was conducted among 924 (91.3%) patients, with only 39 (3.9%) of them being maintained as TIVA, while the remainder 885 (87.5%) were maintained via volatile anesthetics. The demographic data and perioperative data are provided in Table 1. No significant differences were found between those who were satisfied with anesthesia care and those who were not in terms of demographics and perioperative data. Figure 1 depicts a STROBE flow diagram of participant recruitment and selection in the study.

**TABLE 1:** Demographics and general perioperative characteristics.

Characteristics		Satisfaction with anesthesia care		Total (*n* = 1012)	*p*-value
		Dissatisfied (*n* = 24)	Satisfied (*n* = 988)		
Age (years)		43 [31.5–53.8]	45 [31.3–60]	45 [31.3–60]	0.478
Gender	Female	10 (41.7)	494 (50)	504 (49.8)	0.42
	Male	14 (58.3)	494 (50)	508 (50.2)	
Highest attained educational level	Diploma	0 (0)	109 (11)	109 (10.8)	0.263
	Elementary school	1 (4.2)	38 (3.8)	39 (3.9)	
	Postgraduate university degree	2 (8.3)	31 (3.1)	33 (3.3)	
	Secondary school education	6 (25)	294 (29.8)	300 (29.6)	
	University degree	15 (62.5)	516 (52.2)	531 (52.5)	
ASA classification	I	11 (45.8)	313 (31.7)	324 (32)	0.335
	II	11 (45.8)	557 (56.4)	568 (56.1)	
	III	2 (8.3)	118 (11.9)	120 (11.9)	
Type of anesthesia	General anesthesia	23 (95.8)	901 (91.2)	924 (91.3)	0.715
	Other modalities	1 (4.2)	87 (8.8)	88 (8.7)	
Type of surgery	Laparoscopic and minimally invasive	12 (50)	416 (42.1)	428 (42.3)	0.352
	Open general and specialized surgeries	6 (25.0)	179 (18.1)	185 (18.3)	
	Head, neck, and dental surgeries	5 (20.8)	373 (37.8)	378 (37.4)	
	Minor orthopedics and neurosurgical procedures	1 (4.2)	20 (2.0)	21 (2.1)	

*ASA, American Society of Anesthesiologists Physical Status Classification Score; ENT, ear, nose and throat surgery. **Numbers are represented as number (percent) or median [interquartile range].

Internal consistency reliability of the satisfaction scale was assessed in the main study cohort and yielded a Cronbach’s α of 0.74, indicating acceptable reliability.

Upon performing the postoperative follow-up, we found that the most commonly reported postoperative complications was pain, which was reported by 386 (38.1%) patients, followed by sore throat which was reported by 239 (23.6%) patients, and thirdly by nausea being the complaint of 171 (16.9%) patients. An intraoperative adverse event was described in 38 (3.8%) of cases. Notably, only 295 (29.2%) patients did not report any postoperative complications (Table 2).

**TABLE 2:** Postoperatively reported adverse events.

Characteristics		Satisfaction with anesthesia care		Total (*n* = 1012)**	*p*-value Dissatisfied (*n* = 24)
		Dissatisfied (*n* = 24)**	Satisfied (*n* = 988)**		
Intraoperative adverse events		1 (4.2)	37 (3.7)	38 (3.8)	0.605
Postoperative adverse events		20 (83.3)	697 (70.5)	717 (70.8)	0.173
The reported postoperative adverse events	Pain	9 (37.5)	377 (38.2)	386 (38.1)	0.948
	Nausea	6 (25)	165 (16.7)	171 (16.9)	0.273
	Vomiting	2 (8.3)	17 (1.7)	19 (1.9)	0.072
	Dizziness	1 (4.2)	125 (12.7)	126 (12.5)	0.347
	Headache	2 (8.3)	91 (9.2)	93 (9.2)	1
	Anxiety	1 (4.2)	64 (6.5)	65 (6.4)	1
	Eye burning or itching	0 (0)	29 (2.9)	29 (2.9)	1
	Blurred vision	0 (0)	19 (1.9)	19 (1.9)	1
	Eye redness	1 (4.2)	11 (1.1)	12 (1.2)	0.251
	Shoulder pain	0 (0)	6 (0.6)	6 (0.6)	1
	Back pain	0 (0)	2 (0.2)	2 (0.2)	1
	Numbness/paresthesia	0 (0)	11 (1.1)	11 (1.1)	1
	Itchiness	2 (8.3)	9 (0.9)	11 (1.1)	0.026
	Shivering	2 (8.3)	46 (4.7)	48 (4.7)	0.317
	Urinary retention	2 (8.3)	66 (6.7)	68 (6.7)	0.673
	Distension	0 (0)	3 (0.3)	3 (0.3)	1
	Dental complications	0 (0)	6 (0.6)	6 (0.6)	1
	Cough	3 (12.5)	65 (6.6)	68 (6.7)	0.215
	Sore throat	9 (37.5)	230 (23.3)	239 (23.6)	0.105
	Difficulty breathing	1 (4.2)	55 (5.6)	56 (5.5)	1
	Hemoptysis or epistaxis	1 (4.2)	41 (4.1)	42 (4.2)	1
	URTI-like symptoms	0 (0)	7 (0.7)	7 (0.7)	1
	None	4 (16.7)	291 (29.5)	295 (29.2)	0.173
Who addressed postoperative adverse events	Anesthesiologist	6 (25)	169 (17.1)	175 (17.3)	0.645
	Surgeon	0 (0)	12 (1.2)	12 (1.2)	
	Nurse	7 (29.2)	404 (40.9)	411 (40.6)	
	I did not complain	10 (41.7)	383 (38.8)	393 (38.8)	
	None (complain not addressed)	1 (4.2)	20 (2)	21 (2.1)	

**Numbers are represented as number (percent).

Upon investigating Perioperative satisfaction with different anesthesia care and pain management (Table 3), we found significantly lower PACU approach rating on the 10-point Likert scale among those dissatisfied with anesthesia care, with a rating of 6.5 [5.8–9] among those dissatisfied, compared to 8 [8–9] among satisfied patients (*p* < 0.001). In addition, they had significantly higher pain score upon discharge from PACU, with a score 3 [2–4] out of 10, compared to a score of 2 [1–3] among satisfied patients (*p* = 0.033).

**TABLE 3:** Perioperative satisfaction, pain assessment practices, and postoperative pain management characteristics stratified by satisfaction with anesthesia care.

Characteristics		Satisfaction with anesthesia care		Total	*p*-value
		Dissatisfied	Satisfied	(*n* = 1,012)**	
		(*n* = 24)**	(*n* = 988)**		
Rating the approach in the Pre-anesthesia clinic visit		8 [6.3–9]	8 [8–9]	8 [8–9]	0.28
Rating the approach in the holding area at the operating theaters floor		8 [7–9]	8 [7–9]	8 [7–9]	0.09
Remembers the time spent at the PACU after surgery*		22 (91.7)	935 (94.6)	957 (94.6)	0.379
Please rate the approach in the PACU in case you remember this period (*n* = 946)*		6.5 [5.8–9]	8 [8–9]	8 [8–9]	< 0.001
Pain score upon discharge from PACU*		3 [2–4]	2 [1–3]	2 [1–3]	0.033
Pain score at 6 h postoperative		2 [1–2]	1 [1–2]	1 [1–2]	0.087
When was their pain assessed	Only upon complaining	20 (83.3)	500 (50.6)	520 (51.4)	0.002
	Regular reassessment	4 (16.7)	488 (49.4)	492 (48.6)	
How was the assessment done	Just by asking whether they have pain or no	21 (87.5)	814 (82.4)	835 (82.5)	0.785
	NRS-11 or VAS	3 (12.5)	174 (17.6)	177 (17.5)	
Who addressed their pain in the wards	Anesthesiologist	6 (25)	192 (19.4)	198 (19.6)	0.056
	Surgeon	3 (12.5)	36 (3.6)	39 (3.9)	
	Nursing team	15 (62.5)	760 (76.9)	775 (76.6)	
They believe that analgesia was received as prescribed		20 (83.3)	573 (58)	593 (58.6)	0.013
Felt that the pain was being under-treated		6 (25)	70 (7.1)	76 (7.5)	0.007
Felt that they were viewed as malingering or drug seeker		1 (4.2)	9 (0.9)	10 (1)	0.214
Satisfaction with postoperative pain management		7 [6–8.5]	8.5 [8–9]	8 [8–9]	< 0.001
Overall satisfaction with hospital care		7 [6–9]	9 [8–9]	9 [8–9]	< 0.001
Would recommend the hospital for future elective surgeries		21 (87.5)	979 (99.1)	1000 (98.8)	0.002

*PACU, post-anesthesia care unit; NRS-11, 11-point numerical rating scale; visual analog scale.

**Numbers are represented as number (percent) or median [interquartile range].

Postoperative pain was assessed only upon patient complaint in 520 patients (51.4%), while regular reassessment was reported in 492 patients (48.6%). This practice was more frequently reported among patients dissatisfied with anesthesia care compared with satisfied patients (*p* = 0.002; Table 3). Pain assessment was performed primarily by simple inquiry about the presence of pain in 835 patients (82.5%), and postoperative pain management was most commonly provided by the nursing team (76.6%), followed by anesthesiologists (19.6%) and surgeons (3.9%). Overall, 593 patients (58.6%) believed analgesia was administered as prescribed, although dissatisfaction with anesthesia care remained associated with lower satisfaction with postoperative pain management [median score 7 (IQR 6–8.5) vs. 8.5 (IQR 8–9); *p* < 0.001]. Perceived under-treatment of pain was reported by 76 patients (7.5%) and occurred more frequently among dissatisfied patients (*p* = 0.007).

Further analysis of the 10-point Likert scale for overall satisfaction with hospital care revealed significantly lower scores among patients dissatisfied with anesthesia care compared to those who were satisfied [median 7 (IQR 6–9) vs. 9 (IQR 8–9); *p* < 0.001, Mann–Whitney U test]. Willingness to recommend the hospital to family or friends was also strongly associated with satisfaction level. Among satisfied patients, 979 (98.8%) indicated they would recommend the institution, compared with only 21 (87.5%) of dissatisfied patients (*p* = 0.002, chi-squared test; Table 3).

Overall satisfaction with hospital care showed significant positive correlations with satisfaction in perioperative domains. Conversely, satisfaction was negatively correlated with pain intensity at PACU discharge and at 6 h postoperatively (Table 4).

**TABLE 4:** Pearson’s r correlation between satisfaction with anesthesia care and the overall satisfaction with hospital care.

Variable	Pearson’s r in correlation with overall satisfaction with hospital care (*n* = 1,012)*	*p*-value
Please rate the approach in the Pre-anesthesia clinic visit	0.237	< 0.001
Please rate the approach in the holding area at the operating theaters floor	0.410	< 0.001
Please rate the approach in the PACU in case you remember this period (*n* = 946)	0.492	< 0.001
Pain score upon discharge from PACU	-0.225	< 0.001
Pain score at 6 h postoperative	-0.172	< 0.001
Satisfaction with postoperative pain management	0.644	< 0.001

*Pearson’s r, Pearson correlation coefficient.

To address potential threshold sensitivity, we conducted additional analyses using stricter cutoffs ( ≥ 7, ≥ 8, and ≥ 9). The independent associations with PACU approach, regular pain assessment, and perceived under-treatment remained consistent across all thresholds, check (Table 5).

**TABLE 5:** Key findings from sensitivity analyses.

Cutoff	Satisfied (%)	Dissatisfied (n)	PACU approach OR (95% CI)	Regular pain assessment OR	Under-treated OR
≥ 6 (Original)	97.6%	24	1.71 (1.31–2.22)	3.77 (1.17–12.21)	0.31 (0.10–0.92)
≥7	86.0%	142	1.68 (1.45–1.95)	2.85 (1.72–4.72)	0.28 (0.15–0.52)
≥ 8	68.9%	315	1.62 (1.45–1.81)	2.41 (1.68–3.45)	0.35 (0.22–0.55)
≥9	37.7%	630	1.55 (1.40–1.71)	2.12 (1.55–2.90)	0.42 (0.29–0.61)

Multivariable binary logistic regression analysis was performed to identify factors independently associated with satisfaction with anesthesia care (dependent variable: satisfied vs. dissatisfied; Table 6).

**TABLE 6:** Multivariable regression analysis for factors affecting the satisfaction with anesthesia care.

Variable	OR	95% CI for OR*		*p*-value
		Lower	Upper	
Please rate the approach in the pre-anesthesia clinic visit	1.05	0.86	1.30	0.613
Please rate the approach in the holding area at the operating theaters floor	0.96	0.70	1.33	0.819
Please rate the approach in the PACU in case you remember this period	1.71	1.31	2.22	0.000
Pain score upon discharge from PACU	1.05	0.73	1.50	0.809
Pain score at 6 h postoperative	1.23	0.75	2.02	0.404
When was their pain assessed (regular assessment)	3.77	1.17	12.21	0.027
They believe that analgesia was received as prescribed	0.42	0.13	1.33	0.140
Felt that the pain was being under-treated	0.31	0.10	0.92	0.035
Constant	0.52			0.677

*OR, odds ratio; 95% CI, 95% confidence interval for odds ratio; PACU, post-anesthesia care unit.

The overall model was significant (*p* < 0.001). We found that the higher score of the approach at PACU [OR = 1.7; 95% CI: (1.31–2.22); *p* < 0.001], and regular pain assessment (OR = 3.77; 95% CI = 1.17–12.21; *p* = 0.027) were positively and independently associated with satisfaction with anesthesia care, while negative independent correlation was found with under-treatment of pain (OR = 0.31; 95%CI = 0.10–0.92; *p* = 0.035).

Due to the relatively small number of dissatisfied patients (*n* = 24), we performed Firth’s penalized logistic regression as a sensitivity analysis to mitigate potential overfitting. This analysis confirmed that better PACU staff approach (OR 1.68, 95% CI 1.29–2.18, *p* < 0.001), regular pain assessment (OR 3.45, 95% CI 1.12–10.62, *p* = 0.031), and perceived under-treatment of pain (OR 0.33, 95% CI 0.11–0.98, *p* = 0.046) remained independent predictors of satisfaction. The independent associations remained robust across all sensitivity analyses. Model performance was strong (AUC = 0.89), with no evidence of multicollinearity (VIF < 2.5) and good calibration (Hosmer-Lemeshow *p* = 0.312).

## Discussion

The present findings should be interpreted within the broader context of contemporary ambulatory anesthesia, which has shifted toward Enhanced Recovery After Surgery (ERAS) principles adapted for day-case procedures. ERAS pathways in ambulatory settings emphasize multimodal, opioid-sparing analgesia, proactive pain assessment, and optimized perioperative communication—elements that align closely with our independent predictors of satisfaction (PACU staff approach and regular pain assessment) (3, 4, 17).

Our observation that regular pain assessment and attentive PACU care strongly predict satisfaction reinforces the value of structured ERAS-like protocols even in resource-limited settings. Similarly, the association between perceived under-treatment and dissatisfaction highlights the importance of patient-centered pain management and effective communication, consistent with recent literature emphasizing PROMs in ambulatory surgery (5).

Patients satisfaction rates were high and comparable to studies conducted and published worldwide (18). However, such high satisfaction results should be interpreted cautiously; as these rates may be inflated by social desirability bias, whereby patients tend to please medical staff and believe that the staff are doing their best (19, 20). Our sample had a large number of patients with ASA classification II (51.6%). Patient satisfaction was independent of the demographics and Perioperative data. This is in contrast to other studies that found strong correlation between patient satisfaction and the age group, as older age groups had higher satisfaction rates (9, 12, 21). Most postoperative complaints were pain, sore throat, and nausea; which correlates to another study that had pain, nausea, and vomiting as the main postoperative complaints (22). The major finding in this study is that lower satisfaction rates was highly associated with increased postoperative pain score. this aligns with a previous research that lists postoperative pain as a vital factor in overall patient satisfaction (9, 23). In this study, approximately half of patients (51.4%) reported that their pain was addressed only when complaining and not on regular intervals. And when addressed they were asked whether they had pain or not, disregarding the degree of pain. This in turn affected their degree of satisfaction. Interestingly, patients dissatisfied with anesthesia care were more likely to report that analgesia was administered as prescribed (83.3% vs. 58%, *p* = 0.013). This may reflect higher pain intensity and expectations in this group, leading to more frequent requests and administration of analgesics, yet still resulting in perceived inadequate pain relief or poorer communication.

Patient satisfaction strongly influenced overall perception of hospital care and willingness to recommend the hospital to others. The more satisfied the patient was, the more likely they will recommend ambulatory surgery at the hospital to their acquaintances, which was the majority of satisfied patients. Pre-anesthesia clinic, approach at the holding area, and approach at the PACU exerted a major role in the overall satisfaction with hospital care. Similarly another study produced the same result in this aspect. (7)

## Recommendations

A- Implement structured regular pain assessment protocols in PACU and wards.
B- All patients should undergo standardized pain assessment at regular intervals rather than only upon patient complaint.
C- Enhance PACU staff communication and empathy training.
D- Hospitals should introduce targeted training programs for PACU nurses and anesthesiologists focusing on empathetic communication, active listening, and clear explanation of pain management plans.
E- Adopt proactive multimodal and opioid-sparing pain management.
F- Move from reactive (“on-demand”) analgesia to scheduled multimodal regimens (acetaminophen + NSAIDs + regional blocks when feasible) with clear patient education about expected pain levels and available treatments. Special attention should be given to managing patient expectations and promptly addressing perceived under-treatment, which was independently associated with dissatisfaction.
G- Preoperative patient education and expectation setting, by strengthening pre-anesthesia clinic visits to include realistic information about postoperative pain and the pain management plan. This may reduce the gap between expected and experienced pain, thereby decreasing the perception of under-treatment.
H- Quality improvement monitoring by routinely tracking patient satisfaction, pain scores, and perceived under-treatment as key performance indicators in ambulatory surgery units, with regular feedback to frontline staff.

## Limitations

This study has several limitations:

- First; patients with an ASA classification above III were systemically excluded from having an ambulatory procedure due to safety concerns regarding anesthetizing these patients in a ambulatory setting. Consequently our cohort lacked higher risk patients and that reduced generalizabilty of our finding among this group.
- Second; the heterogeneity of the surgical procedures types causes different patterns of recovery and pain outcomes which reflects on patients satisfaction and that was not fully accounted for in the analysis.
- Third; the majority of the surgeries were conducted under general anesthesia limiting our ability to adequately compare results with other modalities and anesthetic techniques.
- Fourth, the relatively small number of dissatisfied patients (*n* = 24) limits the precision of multivariable modeling and raises the possibility of overfitting. Although the main findings were robust in sensitivity analyses using variable reduction and Firth’s penalized regression, simplified modeling, and comprehensive diagnostics. results should be interpreted with caution and ideally confirmed in larger multicenter studies.
- Fifth, we excluded patients with a discrepancy > ± 2 points between self-reported and documented pain scores to minimize reporting bias. While this improves data quality, we recognize that such discrepancies may represent clinically important cases of under-treated pain or communication breakdown. Sensitivity analysis including these patients showed that the main predictors of satisfaction remained consistent, though we cannot completely rule out selection bias.
- Sixth, the questionnaire was administered by second-year anesthesia residents. Although interviewers were trained and independent of the clinical team caring for the patient, we cannot exclude the possibility of social desirability and courtesy bias. Patients may have provided more positive responses due to the presence of healthcare personnel affiliated with the hospital. This bias is well-recognized in patient satisfaction research, particularly in cultures that emphasize respect for authority figures. Future studies should consider using fully independent interviewers or anonymous self-administered electronic questionnaires to minimize this limitation.
- Seventh, Surgery duration was not recorded in this study. As longer procedures are typically associated with greater postoperative pain and recovery challenges, the absence of this variable may limit the interpretation of our results.
- Finally; data were collected from a single center, which means that the results may not fully reflect patient satisfaction in all health care institutions in the country.

## Conclusion

In conclusion, this large single-center study demonstrates that patient satisfaction following ambulatory surgery is highly dependent on effective postoperative pain management, regular pain assessment, and compassionate care in the post-anesthesia care unit. These findings support the adoption of proactive, ERAS-aligned strategies even in resource-constrained settings. Future multicenter studies should evaluate the impact of structured multimodal analgesia protocols and preoperative patient education on satisfaction and recovery outcomes.

In the future we can focus on these deficiencies to improve patient satisfaction and decrease complications. This can be a leading study for further studies that encompass a broader section of the health care system in the country.

## Data Availability

The raw data supporting the conclusions of this article will be made available by the authors, without undue reservation.
